# Smartphone and Web 2.0 tools for CPD amongst General Practitioners in Wessex. A qualitative review

**DOI:** 10.15694/mep.2018.0000155.2

**Published:** 2018-09-10

**Authors:** Antony Willman

**Affiliations:** 1 SMO Bulford Garrison

**Keywords:** smartphone, web 2.0, medical education, general practice

## Abstract

This article was migrated. The article was marked as recommended.

There is increasing ownership of smartphones in the UK. These provide a portable and convenient way to access the Internet and to run a variety of applications. Amongst under- and post-graduate students they are used increasingly for educational purposes. Despite widespread ownership, barriers may exist preventing General Practitioners from using their smartphones for education. This service evaluation confirmed there is high smartphone ownership amongst GPs with a sizeable cohort using them for medical education and CPD. There remains, however, a reluctance to use Web 2.0 tools for CPD.

## Introduction

Most adults in the UK own a smartphone, with some estimates as high as 90% with over ( OFCOM, 2014). While most use relates to gaming and social media activities, there is increasing educational use, in particular in healthcare professions ( Cook *et al.*, 2008). Key enablers of this are smartphones and tablets which in turn enhance mobile learning (m-learning) opportunities. Technical limitations of smartphones, for example, screen size, have been highlighted as a reason for non-adoption ( Gagnon *et al.*, 2012). However, modern devices can run complex software (apps), have larger displays and fast access to the Internet (via 4G and Wi-Fi networks) increasing their value as an educational tool. Medical professionals and medical students have high smartphone ownership rates ( Patel *et al.*, 2015). The use of smartphones for education and use of medical apps is increasing amongst medical students and junior doctors ( Payne, Wharrad and Watts, 2012).

There is no precise definition of Web 2.0. The term was first used in 1999 to describe how the ‘web’ was moving from a static browser window to a more interactive process ( DiNucci, 1999). The term Web 2.0 became established after the first eponymous conference in 2004 “O’Reilly Media and MediaLive”. Web 2.0 can be summarised as a collection of technologies - Instant Messaging, Blogs, Social Networks, Wikis and Podcasts - which allow the user to interact with both the website and other users (network) ( Cervinschi and Butucea, 2010).

The use of Web 2.0 tools in medical education has increased ( Maged Boulos, Maramba and Wheeler, 2006). These tools are used increasingly in a blended learning environment to provide both under, and post-graduate education. Blended learning environments are associated with increased student satisfaction and improvement in individual educational outcomes, e.g. in Problem Based Learning ( Woltering *et al.*, 2009). However, their use as educational tools for CPD is less prevalent. A comprehensive 2007 survey of BMA members demonstrated widespread knowledge but reduced uptake ( Sandars and Schroter, 2007). Four themes were identified: Interested in use but need further training; Barriers due to learning & technology preferences; Quality of resources; Organisational issues. GPs were less likely than doctors in training, consultants and medical students to use a smartphone or Web 2.0 tool for medical education.

There remains a requirement for GPs to undertake CPD for their development, appraisal and revalidation ( RCGP, 2012). With the cost of attending workshops or study days rising and widespread under-manning of General Practice making it increasingly difficult to leave the surgery, supporting CPD in the workplace is essential. GPs often cite time pressures affecting attendance at meetings and the ability to undertake education. There is evidence GPs are less likely to follow guidelines if under time pressure ( Tsiga *et al.*, 2013). Initiatives have mitigated some of this through small group learning with improved learning outcomes, better adherence to guidelines as well as increased learner satisfaction ( Macvicar *et al.*, 2006). Anecdotal and some research evidence suggests GPs are poor adopters of m-learning and Web 2.0 tools, despite the opposite being true of medical students ( Koehler *et al.*, 2012). This service evaluation aimed to explore this using qualitative methods.

## Methods

A Survey Monkey questionnaire with both closed and open free text questions was devised. The closed questions were imported into SPSS for demographic analysis. The free text responses were thematically analysed using Nvivo. Practice Based Small Group Learning (PBSGL) members and GPSTs were chosen as there was a database available. It also represented those who might have a higher uptake of smartphone use for education and social media given they were already using blended learning environments.

## Results/Analysis

The questionnaire was sent out to members of PBSGL groups and GPSTs in Wessex. There were 61 respondents from 253 invitees. 98% had smartphones, 77% used smartphones for medical apps, 65% used smartphones for medical education, 69% used smartphones for social media and 20% used social media for medical education. The breakdown by role is summarised in table 1.

**Table 1.** Smartphone Use, Use for Medical Apps, Use for Medical Education, Social Media for Medical Education and Role Cross tabulation

Role		GP	GP Trainee	GP Trainer	Total _(%)_	%
Smartphone Owner	Yes	45 _(98)_	9 _(100)_	6 _(100)_	60 _(98)_	98
	No	1 _(2)_	0 _(0)_	0 _(0)_	1 _(2)_	2
Total		46	9	6	61	
Smartphone use for Medical Apps	Yes	36 _(78)_	9 _(100)_	2 _(33)_	47 _(77)_	77
	No	9 _(20)_	0 _(0)_	4 _(67)_	13 _(21)_	21
	N/A	1 _(2)_	0 _(0)_	0 _(0)_	1 _(2)_	2
Total		46	9	6	61	
Smartphone use for Medical Education	Yes	30 _(65)_	8 _(89)_	2 _(33)_	40 _(65)_	65
	No	15 _(33)_	1 _(11)_	4 _(67)_	20 _(33)_	33
	N/A	1 _(2)_	0 _(0)_	0 _(0)_	1 _(2)_	2
Total		46	9	6	61	
Smartphone use for Social Media	Yes	31 _(67)_	8 _(89)_	3 _(50)_	42 _(69)_	69
	No	14 _(30)_	0 _(0)_	3 _(50)_	17 _(28)_	28
	N/A	1 _(2)_	1 _(11)_	0 _(0)_	2 _(3)_	3
Total		46	9	6	61	
Social Media for Medical Education	Desktop and mobile	6 _(13)_	2 _(22)_	1 _(17)_	9 _(15)_	15
	Desktop only	2 _(4)_	1 _(11)_	0 _(0)_	3 _(5)_	5
	No	38 _(83)_	6 _(67)_	5 _(83)_	49 _(80)_	80
Total		46	9	6	61	

Thematic analysis of free text responses gave a yield of 364 codable text entries. These were amalgamated into 58 nodes which were grouped into 21 minor themes in 8 categories (Annex A). These are summarised below with some of the coded entries:

**Accessing learning**- the convenience balanced against some access difficulties. Lack of Wi-Fi and poor mobile signal.

*‘For busy GPs, I would see no major disadvantages if the delivery vehicle is good and allows appropriate interaction and feedback.*’ - GP, Male

*‘effective 3G/4G connection cannot be guaranteed - this is frustrating when using m-learning that relies on the internet (as many / most do)’*- GPST, Male

**Educational impact of online and m-learning**- the benefits of GP training were identified as was the diversity. Addressing true learning needs may not occur though.

*‘Good for trainee communication and trainer communication and forums.’*- GP Trainer, Male

*‘too much convenience can mean postponing courses for too long’*- GP, Male

**Educational networking**- a massive online community of healthcare professionals and educators easily accessed was identified. It was felt this may be driven by an informed few however and there is a cohort of non-IT literate healthcare professionals not making full use of the potentials.

*‘Advantage is that it can connect people from all over the world’*- GP, Male

*‘Disadvantages are that less computer literate people may not have the confidence to access’*- GP, Female

**Learning involves social interaction with peers and mentor**- while there was some recognition that online social interaction does occur, the vast majority of respondents reported the benefits of human contact at meetings as well as peer to peer and expert to peer learning.

*‘Disadvantages- no interaction with other course delegates, no ‘networking’ and sharing of ideas’*- GP, Female

*‘loss of that personable approach and ability to mingle with fellow GPs. Just becomes less sociable and everyone is living virtual lives. Almost counter-intuitive to what we try to promote as good communication to our patients.’*- GP Trainer, Female

**Physical constraints of mobile devices**- the balance of portability against the physical limitations of small screen size were recognised. Most GPs preferred working on a computer. The use of mobile learning diaries was identified though.

*‘I use a tablet. Easy to record CPD and notes directly into learning diary.’*- GP, Female

*‘Not often - I find it impractical. I use my tablet more, but mainly use my laptop & desktop’*- GPST, Male

**Signposting to guidelines and learning via the Internet**- there was a positive view that social media and e-mails via mobile devices signposted to new guidelines and journal articles. Concerns were expressed about the ‘echo chamber’ effect, educational rigour of some sites and Internet security.

*‘While I have no concerns/problem with DCAs using social media to highlight & signpost useful resources / recent speciality developments, I don’t see a huge advantage in trying to embed formal academic learning into social media’*- GP, Male

*‘Can’t always easily check sources or know how authoritative info is. Risks of the ‘echo chamber effect’*- GP, Male

**Technical challenges of online and m-learning** - a lot of respondents wanted further training, either peer to peer or as a passive learning event. Uptake was clouded by previous poor experiences of IT training.

*‘Local GP teaching to help recommend suitable sources/apps. Short teaching session in surgery to show us how to use.’*- GP, Female

*‘Would be good if regional educational teams ran advice/walk-in sessions to get established with right apps and how to access.... ‘an idiots guide”*- GP Trainer, Female

**Work-life balance**- there was almost universal consensus that mobile devices are already too intrusive and social media for education would only make this worse. There was also a view that work is work, home is home. The majority of respondents cited time factors in poor uptake.

*‘Simply a matter of time available and sometimes feels like yet another modality of learning that we are having to embrace.’*- GP Trainer, Female

*‘I can’t see any use regarding twitter, and frankly, my life is busy enough without introducing a plethora of other lines of communication- which aren’t actually very good at communicating’*- GP, Male

## Discussion

The ownership of smartphones is high reflecting the general population. Use of smartphones for medical education and medical app use is encouraging. From the free text responses, this probably represents better app development and ease of use, the use of medical calculators/algorithms and electronic drug compendiums (e.g. eBNF). The uptake of between 65 and 77% compares favourably to the US (85%) where smartphone use is more established and also linked to billing ( Maged N Kamel Boulos *et al.*, 2014). While the use of smartphones for social media is similar to that for medical education, there is still poor uptake in the use of Web 2.0 tools for medical education.

From the thematic analysis, GPs favour the social interaction and networking that takes place at clinical meetings. They learn from each other (as well as the subject matter expert). They are less inclined to look at Web 2.0 tools as a viable alternative. There is also a concern about security, content and educational rigour. There is a large cohort who do not know how to use Web 2.0 for education.

This is important for several reasons. Examples where Web 2.0 enhance medical education include:

1. the increasing the use of educational Webinars which are often signposted to by a Tweet or e-mail ( Rosengren *et al.*, 2015);

2. Delegates live Tweeting at conferences disseminating links to best practice or updated guidelines;

3. WhatsApp special interest groups asking clinical questions of their peers in a safe and secure environment;

4. Blended learning courses, e.g. The South Coast Dermoscopy Associates, who use a closed online group between teaching sessions for discussion of cases ( Hayes, 2017);

5. Free Online Access Medical Education (‘Meducation’), or FOAMed, provides users with a vast array of medical information and CPD materials.

GPs need the skills and confidence to use these.

With future budgets for medical education likely to be under pressure, the traditional face to face educational meeting may become the exception, not the norm. Workshops introducing GPs to Web 2.0 tools for medical education could be organised alongside the adoption of blended learning for clinical update and refresher courses. Not only would this reduce costs, but the educational impact on CPD would also increase.

## Conclusion

This is a snapshot of a small, purposefully selected population. It does raise some key messages which may be generalisable to the wider GP workforce. There is a training requirement for GPs in how to use social media for education. GPs valued the networking between colleagues at courses and conferences. However, most respondents recognised the future benefits of mobile learning platforms and wanted to engage more. Further, larger surveys may provide a better picture.

## Take Home Messages

• GPs are already adept at using smartphones and mLearning for CPD activities.
• They are less confident in the use of Web 2.0 and Social Media CPD for a number of reasons and future training is required to address this.
• The benefits of attending CPD events is greater than the sum of the parts, being valuable enablers for networking and sharing experiences.
• Future CPD models need to be looking at a blended environment incorporating all of these elements.

## Notes On Contributors

Antony Willman is a GP and GP Trainer working for the MOD in Wiltshire. His interests include medical education and promoting the use of mLearning and Web 2.0 tools for postgraduate and GP CPD.

## Appendices

### Appendix A Nodes, Themes and Categories

**Table:**

Name	References coded
**ACCESSING LEARNING**	93
**Access challenges with on-line and M-learning**	39
App and Portal becomes out of date unless supported	4
App and Portal cost	4
App and Portal difficult to access	11
On-line courses require motivation	3
Social Media can be very time consuming	3
Web 2.0 requires Wi-Fi or 4G network	14
**How accessing on-line and M-learning could be improved**	1
Offline availability	1
**Positive aspects of accessing on-line and M-learning**	53
Ability to repeat on-line learning	2
Ability to study on-line at any time or place	10
Evolving Use of Web 2.0 for Learning	4
Opportunistic as opposed to Study Leave	4
Undertake at own pace	12
Web 2.0 - convenience and ease of access	21
**EDUCATIONAL IMPACT OF ON-LINE AND M-LEARNING**	6
Mobile Platforms useful in GP Training	2
Web 2.0 has a large array of courses and learning portals	2
Web 2.0 may not address true learning needs	1
Web 2.0 useful for large interactive meetings	1
**EDUCATIONAL NETWORKING**	20
**Networking enhanced by the Internet and certain Social Media platforms**	8
Web 2.0 connects people	1
Web 2.0 enhances educational networks	2
Web 2.0 has Networking benefits	5
**Potential risks of Internet based Educational Networking**	12
Strong Online Advocacy probably not representative of majority of GPs	3
Web 2.0 may discriminate against non-IT literate users	9
**LEARNING INVOLVES SOCIAL INTERACTION WITH PEERS AND MENTORS**	61
**Meetings and small group work enhance learning**	23
Networking benefits of clinical meetings	11
Sharing of ideas at meetings	7
Social interaction of clinical meetings	5
**Reduced learning from both peers and experts**	12
Multi-Format Education	2
Web 2.0 lacks face to face expert opinion	10
**Reduced social interaction with on-line learning**	26
Web 2.0 - lacks human interaction & discussion	26
**PHYSICAL CONSTRAINTS OF M-LEARNING DEVICES**	37
**Size of screen and device hinders effective m-learning**	34
Mobile Platforms limited by physical size	31
Prefer physical medium	3
**Small size allows portability and ease of access**	3
Mobile app used for CPD and appraisal	3
**SIGNPOSTING TO GUIDELINES AND LEARNING VIA THE INTERNET**	52
**Concerns about Internet security**	8
Web 2.0 associated with security concerns	8
**Concerns about reliability of education material found on-line**	22
Echo Chamber Effect	6
Web 2.0 has less reliable information	7
Web 2.0 has reduced educational rigor	7
Web 2.0 possibly pharma influenced & therefore biased	2
**Dissemination can be enhanced by use of the Internet and Social Media platforms**	22
Web 2.0 for signposting	10
Web 2.0 useful for learning ‘bites’	3
Web 2.0 useful in PBSGL meeting	1
Web 2.0 useful reference tool	8
**TECHNICAL CHALLENGES OF ON-LINE AND M-LEARNING**	41
**Current technical challenges of on-line and M-learning**	27
Previous poor experience of IT training	1
Unprofessional to use Smartphones in front of patients	1
Web 2.0 easier for those who are IT literate already	4
Web 2.0 requires additional training	18
Web 2.0 tools can be one dimensional	3
**How technical support could be delivered**	14
Peer to peer technical help	3
Practice Based Education Package on Web 2.0 for Education	1
Raising Awareness of Web 2.0 for Education	10
**WORK LIFE BALANCE**	54
**Disadvantages of mobile devices and Social Media**	34
Always on Society perpetuated by mobile devices	5
Not interested in Social Media	4
Time Pressure	6
Too much e-learning already	3
Web 2.0 - information overload	1
Web 2.0 for social activities, not work	9
Work Encroaching on Leisure Time	6
**How on-line learning can affect work life balance**	20
Reduced overheads of online learning	3
Reduced study leave	4
Web 2.0 reduces travelling time	13
