# Supplementary material for: Smartphone and Web 2.0 tools for CPD amongst General Practitioners in Wessex. A qualitative review
Source: MedEdPublish (2016). 2018 Sep 10;7:155. Originally published 2018 Jul 25. [Version 2] doi: 10.15694/mep.2018.0000155.2 (PMC10701844; doi:10.15694/mep.2018.0000155.2)
Supplement: Peer review history

# Reviewer report: Reviewer response for version 2

**DOI:** 10.21956/mep.20222.r31319

**Published:** 2019-03-19

**Authors:** Trevor Gibbs

**Affiliations:** 1 AMEE

This review has been migrated. The reviewer awarded 4 stars out of 5 As we move into the area of Technology Enhanced Learning (TEL) , it is almost inevitable that we incorporate it into the CPD activities of practicing doctors. Although not completely answering all of the questions we need to ask about the value of TEL, this paper begins to structure some important evaluations. I do believe this is the start of a potentially much bigger project by the author and look forward to perhaps reading these new researches in the future. In the meantime I do believe that this paper is worth reading by those involved in the area.

Reviewer Expertise:

NA

No decision status is available

# Reviewer report: Reviewer response for version 2

**DOI:** 10.21956/mep.20222.r31318

**Published:** 2018-12-01

**Authors:** Ken Masters

**Affiliations:** 1 Sultan Qaboos University

This review has been migrated. The reviewer awarded 3 stars out of 5 Although this study is of a very small snapshot, it does give some useful insight into the use of smartphones by GPs in Wessex. While it does not tell us much about the GPs’ actual usage patterns, it does highlight their attitudes towards usage, and possible barriers. This could serve as a useful entry project for wider expansion. So, overall, a bit limited in scope, but a useful read.

Reviewer Expertise:

NA

No decision status is available

# Reviewer report: Reviewer response for version 1

**DOI:** 10.21956/mep.18981.r27195

**Published:** 2018-07-31

**Authors:** Tripti Srivastava

**Affiliations:** 1 Datta Meghe Institute of Medical Sciences Deemed University

This review has been migrated. The reviewer awarded 3 stars out of 5 The authors have analyzed the extent of Web 2.0 usage amongst GPs for Continuous Professional Development. The growing dependence and thereby concerns regarding information authenticity are prime considerations despite Web 2.0 paving the way for interactions and contributions in webspace. The Qualitative analysis reveal the concerns of security, content and educational rigor. The study gives a glimpse of GPs utility concerns and perception about web 2.0, however; there needs to be more in-depth exploration on how the utility can be strengthened in medical practice. In-spite of all limitations, the greatest advantage here is participation in real time at workplace! A more rigorous methodology will be more helpful to bridge the knowledge gap and generate a hypothesis for further studies.

Reviewer Expertise:

NA

No decision status is available

# Reviewer report: Reviewer response for version 1

**DOI:** 10.21956/mep.18981.r27196

**Published:** 2018-07-25

**Authors:** Subha Ramani

**Affiliations:** 1 Harvard Medical School, Brigham and Women's Hospital

This review has been migrated. The reviewer awarded 2 stars out of 5 This article is interesting and well written, and well referenced. Authors are correct that there is increasing ownership of smart phones which are more portable than laptops or even tablet computers, and can potentially provide point-of-care information. Technology and construction of smart phones are also improving and more interactive applications are being created everyday. I do have a few comments: I am not entirely sure how much it advances the field of technology facilitated learning especially in CPD. Smart phones do not belong to one species and they are not of uniform size. The smaller ones which are still owned by many are not as convenient to use for long periods of time as the larger ones. We do not know how many respondents actually owned a large smart phone. Web 2.0 based CPD can probably be more easily accessed via tablets and I am not completely sure why the authors decided to focus on smart phones. Why should anyone use smart phones for interactive CPD when CPD needs time to review, time to prepare, time to respond and that time is not available during regular work hours. Since CPD participation mostly occurs when GPs would be at home, the focus on smart phones is not entirely clear when they have easy access to tablets and laptops or desktops.My main concern is regarding the methodology. The authors state that qualitative analysis of free text comments was performed and thematic analysis conducted. This is reasonable. However, they also report that grounded theory approach was used. I do not believe this is correct. Grounded theory is not synonymous with thematic analysis, generally not performed on brief open text comments, involves content from in-person interviews / focus groups, theoretical sampling, constant comparative analysis and construction of theory. I do not see any of this in this study. The paper would be interesting to all those engaged in designing technology facilitated CPD, but I would recommend that the authors combine smart phones and tablets if they are exploring CPD engagement among GPs. The comparison between social media use and educational use is not immediately relevant to me. One requires relatively superficial and the other much deeper cognitive engagement.

Reviewer Expertise:

NA

No decision status is available
